# Brain meningioma invading and destructing the skull bone: replacement of the missing bone *in vivo*

**DOI:** 10.2478/v10019-011-0036-1

**Published:** 2011-11-16

**Authors:** Tomaz Velnar, Rado Pregelj, Clara Limbaeck-Stokin

**Affiliations:** 1 University Medical Centre Ljubljana, Department of Neurosurgery, Ljubljana, Slovenia; 2 Institute of Pathology, Medical Faculty, Ljubljana, Slovenia

**Keywords:** meningioma, brain, invasion, bone reconstruction

## Abstract

**Background:**

Meningiomas are frequently encountered tumours. In those invading locally into the adjacent tissue, reconstructions may pose a problem.

**Case report:**

We report a case of a benign convexity brain meningioma with invasion into the skull bone and subcutaneous tissue. The tumour was removed completely, together with the infiltrated tissue and the defects were successfully closed with *in vivo* bone reconstruction.

**Conclusions:**

The reconstruction of the skull bone is sometimes needed after the benign meningioma excision. Artificial bone may be a suitable material, allowing fast intraoperative reconstruction with excellent brain protection and cosmetic effect during the one-stage procedure.

## Introduction

In neurosurgical practice, meningiomas are frequently encountered tumours and they represent about 20% to 25% of central nervous system neoplasms.^1^–^3^ Arising from arachnoidal cells lining the brain and spinal cord, they may be found intracranially and intraspinally. In addition, meningiomas may originate as extracranial or extraspinal masses. These types of tumours are referred to as ectopic and have been described in various locations, the most frequent being the head, neck and soft tissue alongside the vertebral column.^1^,^2^,^4^

Meningiomas usually affect middle aged and older adults and contrary to oligodenroglial tumours are twice as frequent in women as in men.^4^,^5^ Progesterone receptors have been found in meningeal tumour cells and possibly this hormone positively influences tumour development and progression. Genetic mutations in the neurofibromatosis 2 gene (NF2), immunological factors and exposure to both high and low dose of ionizing radiation have been recognised among risk factors.^2^

Ninety percent of meningiomas are slow growing and benign tumours, the remaining ones are invasive or truly malignant.^1^ Most meningiomas have good long-term prognosis after the treatment, some display an aggressive clinical behaviour. The vicinity and compression of the eloquent brain zones, venous sinuses, skull base location and adjacent bone destruction may often lead to serious and potentially lethal consequences.^1^,^4^ Clinically, meningiomas are revealed by various symptoms including neurologic deficits and epileptic seizures.^1^ Surgery still remains the principal form of the treatment and must be preceded by appropriate preoperative diagnostics.^6^–^8^

Although the majority of meningiomas behave as expansive lesions, compressing the brain tissue, they may cause erosion on the neighbouring structures, especially bone. It is the location and particularly invasion of tumour into adjacent tissue that may hamper radical resections and reconstructions by simple surgical means.^1^,^9^,^10^ In such cases, the reconstruction of the skull bone is problematic due to tissue deficit. Many alternatives exist, from autografts, allografts or artificial replacement material.^11^,^12^ We report an unusual case of a meningioma of the brain, located in the premotor and motor cortex in the frontoparietal region, invading and destroying the skull bone and subcutaneous tissue. The tumour was removed completely, together with the infiltrated tissue and the defects after the operation were successfully reconstructed with *in vivo* bone reconstruction.

## Case report

A 66-year old gentleman in otherwise good general health was admitted to the neurosurgical department due to a skull deformation in the left frontoparietal region, which was growing progressively. He first noticed it approximately seven months ago and complained of dull headaches, located in the left half of the head that were noticed a few times weekly. No other complaints in connection with his health status were reported at the admission.

The neurological status during the clinical examination was normal. Locally, a skull tumour of 5 cm in diameter was felt. It was immobile and insensitive on palpation and the skin covering it was normal.

**FIGURES 1A, 1B:**
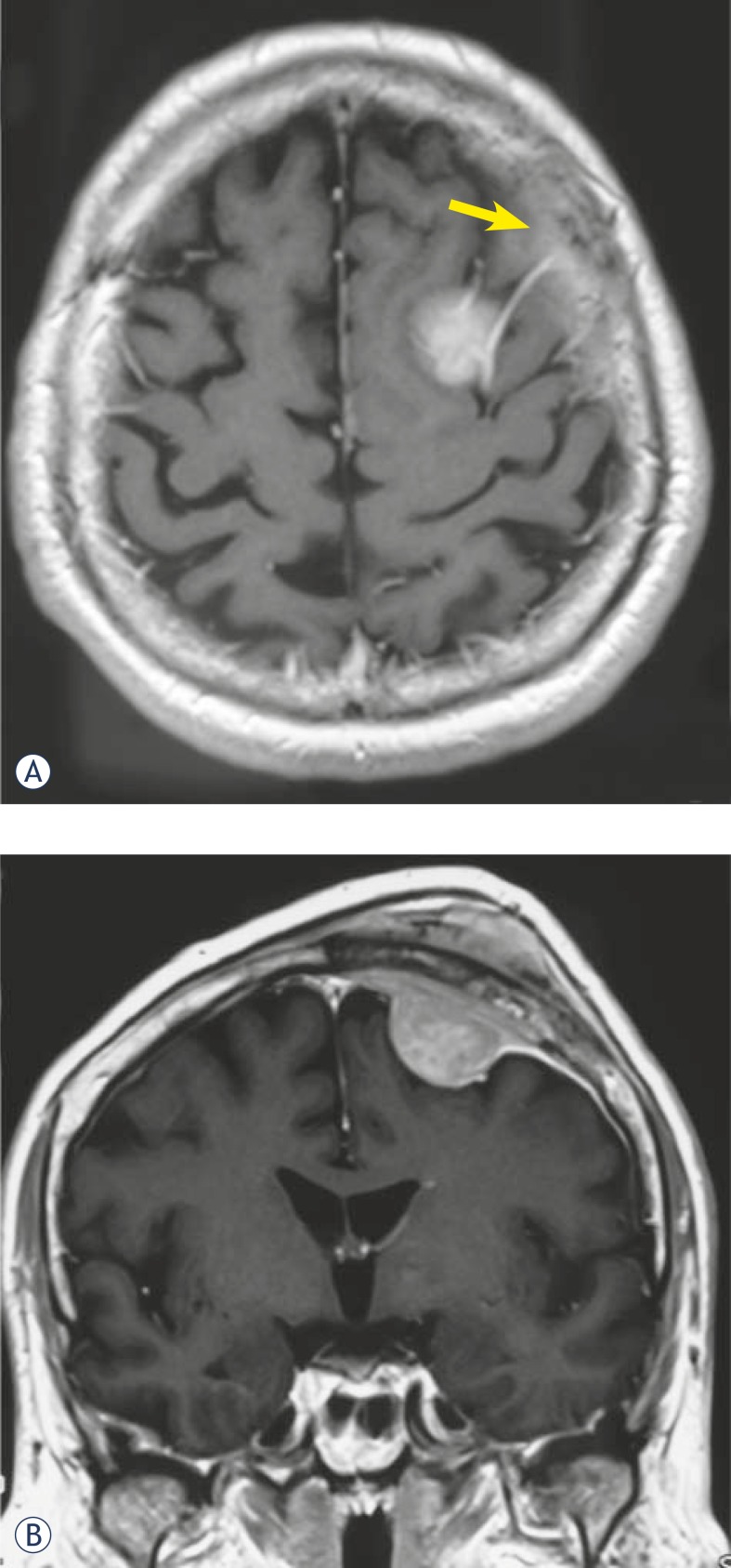
The axial view of the CT scan showing homogenous lesion in the motor/premotor area, radiologically classified as meningioma, with hyperostotic bone (arrow) (A). The coronal view of the tumour, growing intra- and extracranially (B).

The computer tomography (CT) and magnetic resonance (MR) imaging revealed an intracranial expansive lesion of 8 cm in diameter, compressing the cortex and invading the skull bone and subcutaneous tissue (Figures 1A and 1B). The CT angiography did not show any signs about sinus invasion. Surgery was indicated.

**FIGURES 2A, 2B, 2C, 2D:**
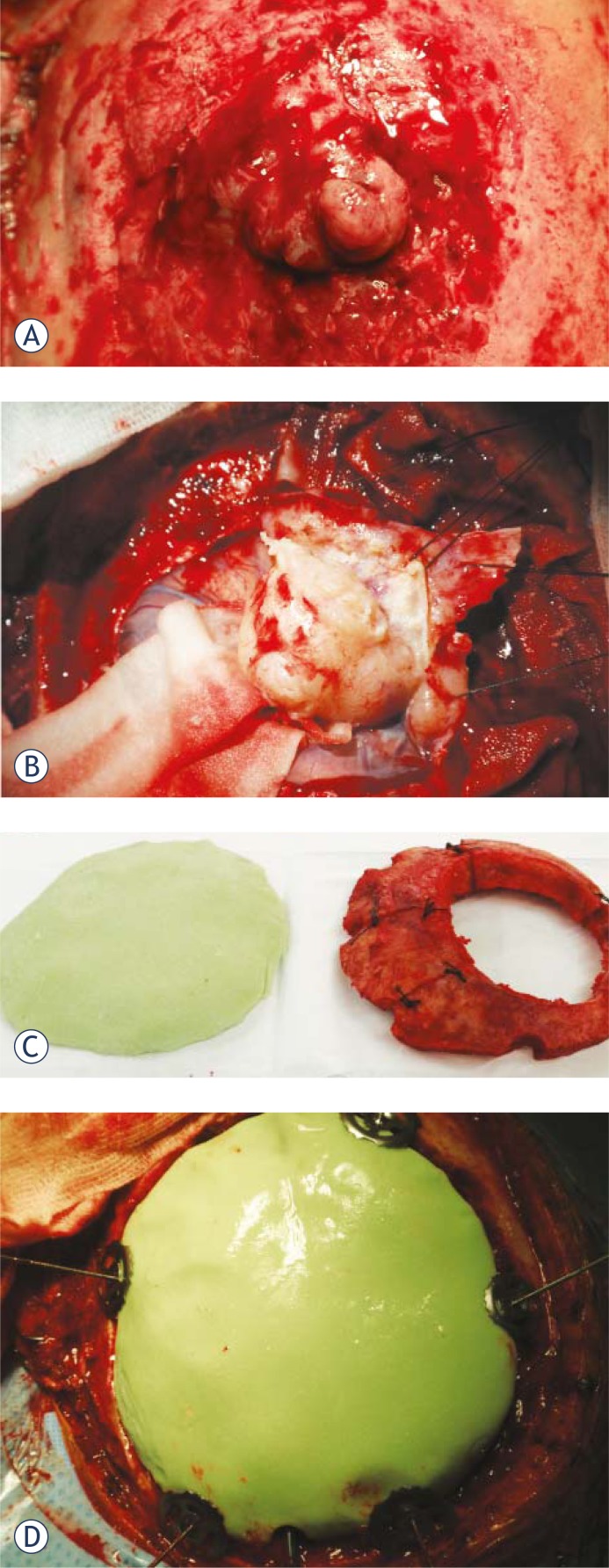
Intraoperative view of the meningioma growing through the skull bone into the periosteum and the subcutis. The scalp has been retracted laterally (A). Removal of the intracranial part of the meningioma: the tumour was dissected from the brain tissue, gently lifted off the brain via suspension and removed together with the infiltrated dura. Special care was taken not to damage the vessels (B). The artificial bone modelling and its template. Green material is polymethylmetacrylate. During the operation, the outer ring of the bone visible on the right was removed first, only then the tumour dissection and removal of the inner bony ring started (C). The artificial bone in place, fixed with titanium clamps at the edge (D).

The operation was performed via midline incision. In the subcutis, the tumour mass growing through the bone was seen, infiltrating the periosteum and galea (Figure 2A). A round section of the skull bone was performed, encircling the tumour first. Then, the bone in the very vicinity of the tumour was drilled in such a way that two circular bone flaps were formed around the tumour as it was not possible to elevate the first bone flap due to the tumour adhesion to the bone without damaging the bridging veins and the dura. The tumour was then microsurgically removed, carefully dissected and elevated off the brain substance (Figure 2B). The tumour origin was in the dural convexity over the left motor and premotor cortex. The cortex was relatively spared, though severely compressed and the superior sagittal sinus was also compressed but otherwise intact. However, the bone was porotic and invaded by the tumour, which spread through the periosteum into the galea. The tumour was completely excised together with all infiltrated extracranial tissue.

**FIGURE 3:**
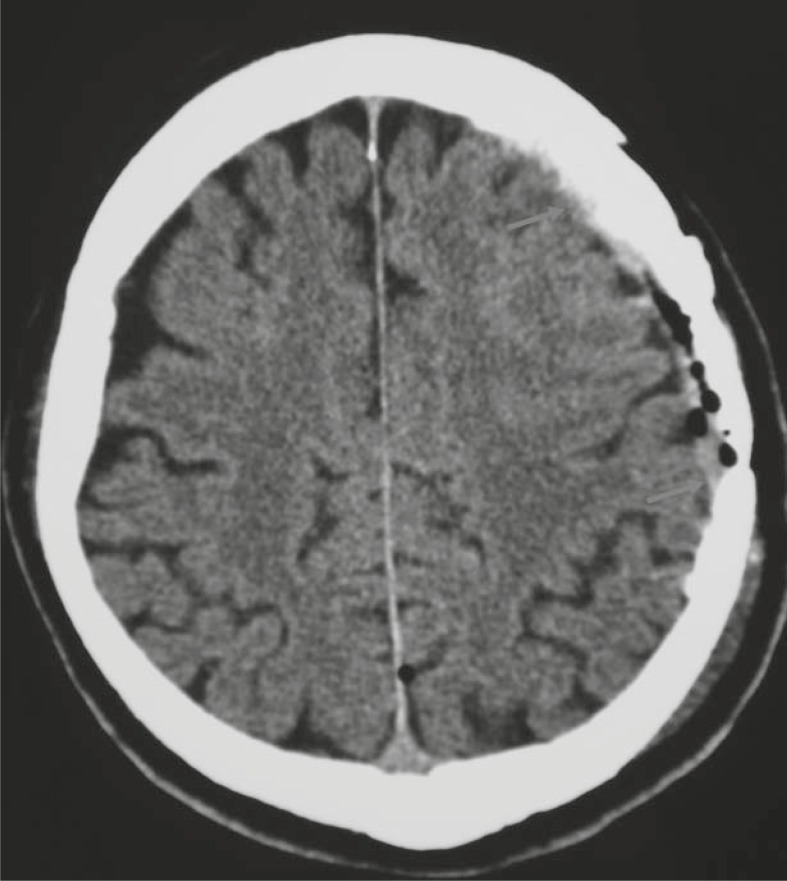
The axial view of the postoperative CT scan with the artificial bone (arrows) covering slightly oedematous brain tissue.

Extensive defects of the dura mater and bone were well reconstructed with dura replacement material (lyophilised dura) and water tightly sealed with fibrin glue in order to avoid liquorrhea. Artificial bone was modelled *in vivo* from two component polymethylmetacrylate material, which was moulded and modelled according to the shape of the removed bone just before closure (Figures 2C and 2D). The original bone flap was used as a template. The fit was very good, giving an excellent cosmetic result as well as brain protection. The new artificial bone flap was fixed to the skull bone with titanium plates. Finally, the wound was closed in layers. After the operation, the patient was neurologically intact. The control CT scan showed a good position of the implant with no fluid collection underneath (Figure 3). The rest of the postoperative course was uneventful.

**FIGURE 4:**
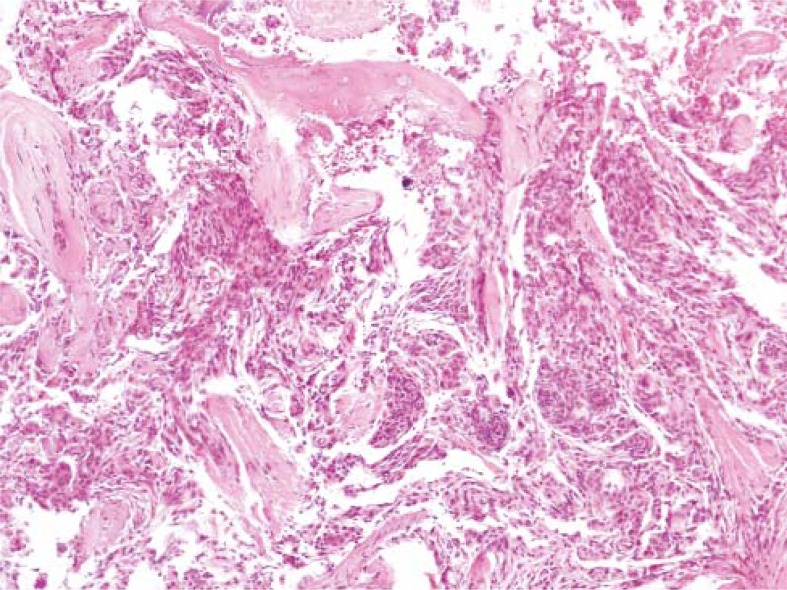
The histological image of the extracranial part of the fibrous meningioma, showing tumour cell invasion into the muscle tissue of the galea.

Histology showed that the tumour was a conventional fibrous meningioma, WHO grade 1. It indeed originated from the dural convexity and spread through the bone into the subcutis (Figure 4). No additional treatment was recommended.

## Discussion

Our patient was operated on for a benign meningioma, which was especially interesting because of the invasion into the skull bone, its destruction and invasion into the subcutaneous tissue as well as the postoperative question about tissue reconstruction. The majority of meningiomas are benign tumours that behave as expansive lesions.^1^,^3^,^10^ Symptoms usually arise due to compression of the brain and erosion of the neighbouring tissue.

Some of meningiomas are invasive and about 5% of meningiomas are malignant, more likely causing direct invasion.^1^,^3^,^13^–^15^ Besides invasive and malignant meningiomas, benign meningiomas may also invade bone. In all cases, the reconstruction of the removed bone is necessary.

Patients with meningiomas are often elderly people with associate diseases that preclude radical resections and complicate a postoperative course. Because of tissue deficit and extensive operation, the reconstruction of the missing tissue, especially the skull bone and soft tissue, is problematic.^16^–^18^ There are many alternatives to repair the missing tissue, nowadays three main techniques are used: autografts, allografts and artificial replacement material.^9^,^10^,^19^ The selection of the material and operative technique depends on surgeons’ experience and preferences in addition to size, location, shape and depth of the bone defect.

About 50% of cases show hyperostosis of the bone overlaying the tumour, with meningothelial tumour cells infiltrating the bone itself.^9^,^10^ A lot of technical difficulties may arise during the operation and because of that, many skull base tumours, principally those of the anterior or middle cranial fossa and those extending into the orbit were not excised completely. Recently, improved techniques of craniofacial surgery have been developed, allowing a wide range reconstruction and leading to more successful clinical result.^19^

In order to accomplish a complete resection, a combined intra- and extracranial resection is required, involving the removal of the hypertrophic bone. It was suggested that strict adherence to oncological principles should be applied also in the case of benign neoplasms in order to prevent contamination of wounds with tumour cells and potential recurrence.^20^ Often, a radical resection may be attained with low morbidity in operated patients, providing a significantly better long-term clinical outcome.^10^ In such extensive resections, the aesthetic reconstruction of large bone defects may pose a significant issue during the operation. Viable tissue in the form of autografts and allografts is one attractive option, another one is artificial replacement material.^9^,^11^,^12^

In this particular case, we decided for *in vivo* reconstruction of the missing bone with artificial replacement material for several reasons. The autografts, which are available in the form of free tissue transfer, rotational flaps and combined as well, allow coverings of large volume tissue defects, especially those of soft tissue.^19^,^21^ Vascularised tissue is less prone to infection and will survive easily than grafts without direct vascular supply. Autografts are safe in terms of disease transmission and exhibit no immune response reactions. Bone grafts show low infection and desorption rate that leads to relatively short term of graft incorporation.^21^ Moreover, vascularised tissue is relatively resistant to postoperative irradiation, which may also become necessary after the resection of grade III and sometimes of grade II meningiomas.^11^,^19^,^21^,^22^ However, to protect healthy tissue, specially brain and vessels, we use limited postoperative irradiated fields in contrast with prophylactic cranial irradiation.^23^

On the other hand, the use of viable tissue has many drawbacks, especially morbidity at the donor site after the operation, higher infection rate due to more extensive operative process, longer intraoperative time and a need of plastic surgeon. When allografts are considered, the infection risk is higher and immunological reactions are possible, complicating the recovery and slowing down the healing process.^21^

Further possibilities are cranioplasty implants. Some are manufactured in advance, according to the shape of the bone defect, other may be modelled intraoperatively from titanium meshes or various composite materials. In our case, we decided for the artificial bone reconstruction. Custom made cranipolasty implants are made in advance according to the shape of the bone defect, therefore, the fit is not always appropriate, increasing the possibilities that the implant may shift. Furthermore, a second operation for the implantation is needed. Our goal was to complete the tumour resection and the reconstruction in one leg, posing less operation-associated risks and enabling faster recovery. We reconstructed the defect from polymethylmethacrylate, which is an efficient and relatively straightforward procedure. It can be performed immediately after the tumour resection, it is fast and yields excellent cosmetic results. After mixing the two components of polymethylmethacrylate, the material is soft and it may be modelled in the shape of the removed bone, exactly filling the bone defect. It hardens in about ten minutes, allowing subtle additional adjustments in shape and fit during modelling. The curvature of the implant may be adjusted according to the curvature of the skull and the implant may be easily fixed with titanium plates and screws, titanium clamps or absorbable clamps, providing good stability. The implant, when in place and air-dried, yields a solid construct that perfectly matches the patient’s natural head shape and has good strength in both compression and tension. Our operative procedure with bone modelling did not pose any particular technical problem and was time saving with good result of the reconstruction.

Among the artificial materials, artificial bone is an efficient alternative to titanium plate or mesh for intraoperative bone reconstruction.^9^ According to our experience, this technique is useful in appropriate conditions, as it is time-saving, straightforward and enables brain protection with good cosmetic result after the extensive cranial vault defect at the time of tumour resection.

## Conclusions

This case illustrates how reconstruction of the skull bone is sometimes needed after the benign meningioma excision. Artificial bone may be a suitable material, allowing fast intraoperative reconstruction with excellent brain protection and cosmetic effect during the one-stage procedure.
